# Outcomes of different anesthesia techniques in nonagenarians treated with mechanical thrombectomy for anterior circulation large vessel occlusion: An inverse probability weighting analysis

**DOI:** 10.1177/23969873241293009

**Published:** 2024-10-30

**Authors:** Viva Levee, Mariarosaria Valente, Francesco Bax, Liqun Zhang, Simona Sacco, Matteo Foschi, Raffaele Ornello, Katherine Chulack, Emma Marchong, Fahad Sheikh, Feras Fayez, Caterina Del Regno, Mohammed Aggour, Massimo Sponza, Francesco Toraldo, Razan Algazlan, Kyriakos Lobotesis, Daniele Bagatto, Nina Mansoor, Dheeraj Kalladka, Vladimir Gavrilovic, Cristian Deana, Flavio Bassi, Berry Stewart, Gian Luigi Gigli, Soma Banerjee, Giovanni Merlino, Lucio D’Anna

**Affiliations:** 1 Department of Stroke and Neuroscience, Charing Cross Hospital, Imperial College Healthcare NHS Trust, London, UK; 2 Stroke Unit, Udine University Hospital, Udine, Italy; 3 Clinical Neurology, Udine University Hospital and DMED, University of Udine, Udine, Italy; 4 Department of Neuroscience, George’s University of London, Stroke, London, UK; 5 Department of Biotechnological and Applied Clinical Sciences, University of L’Aquila, L’Aquila, Italy; 6 Neuroradiology, Udine University Hospital, Udine, Italy; 7 Neuroradiology, Department of Imaging, Charing Cross Hospital, Imperial College Healthcare NHS Trust, London, UK; 8 Department of Anaesthesia and Intensive Care Health Integrated, Agency of Friuli Centrale, Udine, Italy; 9 Department of Anaesthesia, Charing Cross Hospital, Imperial College Healthcare NHS Trust, London, UK; 10 Department of Brain Sciences, Imperial College London, London, UK

**Keywords:** nonagenarians, mechanical thrombectomy, general anesthesia, acute ischemic stroke

## Abstract

**Introduction:**

There is a lack of evidence for the optimal type of anesthesia technique in patients ⩾ 90 years with acute ischemic stroke undergoing mechanical thrombectomy (MT) as this subgroup of patients was often excluded or under-represented in previous trials. We aimed to compare outcomes between general anesthesia (GA) and non-GA techniques in patients ⩾ 90 years with large vessel occlusion (LVO) undergoing MT.

**Patients and methods:**

Our study included patients ⩾ 90 years with anterior circulation LVO, NIHSS ⩾ 6, ASPECTS ⩾ 5 consecutively treated with MT within 6 h after stroke onset in three thrombectomy capable centers between January 1st, 2016 and March 30th, 2023. Inverse probability weighting (IPW) was used to reduce bias by indication of the anesthesia type on study outcomes. We used a weighted ordinal robust logistic regression analysis to explore the primary outcome of modified Rankin Scale (mRS) shift at 90 days in GA versus non-GA treated patients. Secondary outcomes included 90-day mortality, symptomatic intracranial hemorrhage (sICH) and TICI score of 2b, 2c, or 3.

**Results:**

We included 139 patients ⩾ 90 years treated with MT, 62 were in GA group and 77 in non-GA group. There was a significant shift for worse mRS scores at 90-day in non-GA treated patients (cOR 3.65, 95% CI 1.77–7.77, *p* = 0.001). The weighted logistic regression showed that non-GA technique was an independent predictor of 90-day mortality (OR 7.49, 95% CI 2.00–28.09; *p* = 0.003).

**Conclusion:**

Our study indicated that nonagenarians with acute ischemic stroke treated with MT without GA have a worse prognosis than their counterparts undergoing MT with GA. Further studies in larger cohorts are warranted to evaluate the optimal type of anesthesia in this patient population.

## Graphical abstract

**Graphical abstract:**
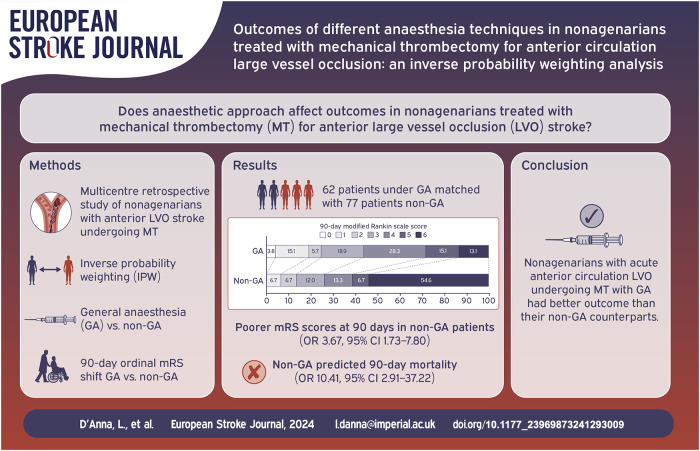

## Introduction

Mechanical Thrombectomy (MT) currently represents the standard of care for acute ischemic stroke due to large vessel occlusion (LVO) in adults regardless of the age of the patient.^1^ Although major clinical trials have demonstrated the benefit of MT for the general population, the degree of benefit is less clear in patients aged 90 years or older as this subgroup was often excluded or under-represented in previous trials.^2–5^ Data obtained from analysis of retrospective cohort registries suggested that MT is generally safe in a selected cohort of nonagenarian patients with acute ischemic stroke due to LVO.^6–12^

MT for LVO stroke is either performed under general anesthesia (GA) or with non-GA techniques such as conscious sedation or local anesthesia alone. A recent systematic review and meta-analysis of seven randomized controlled trials (RCTs) has shown that GA is associated with higher recanalization rates and better functional outcome at 90 days compared to non-GA techniques, mainly conscious sedation, in patients undergoing MT for LVO stroke.^13^ However, this analysis included 988 MT patients with a median age ranging from 60 to 73 years. Therefore, there is still a lack evidence for the optimal type of anesthesia during MT for patients with acute ischemic stroke who are aged 90 and older. Considering the overall aging population and the increasing number of patients with LVO aged 90 years and older who will meet the criteria for MT in the future, it is of paramount importance to investigate the impact of anesthesia type on the clinical outcomes of this age group. Therefore, we sought to compare non-GA and GA in terms of clinical outcomes among patients aged 90 years and older with LVO undergoing MT.

## Patients and methods

### Study design and patients

This is a multicenter, observational, investigator-initiated, retrospective study, that included all acute stroke patients aged 90 years or older consecutively treated with MT in three thrombectomy capable centers: Charing Cross Hospital, Imperial College Healthcare NHS Trust, London (UK); St George’s University of London, London (UK); Udine University Hospital, Udine (Italy) between January 1st, 2016 and 30th March 2023 with local stroke registries available.^14–16^ The study was conducted in accordance the most recent version of the Declaration of Helsinki. We compared patients treated with GA (GA group), defined by endotracheal intubation, versus those treated without GA (non-GA group), the latter including conscious sedation or no sedation at all. Patients who began the procedure with non-GA and converted to GA during the procedure were assigned to the GA group. The choice of type of anesthesia was not carried out according to a specific study protocol but according to the practice of the treating anesthetic consultant.

### Patient inclusion and exclusion criteria for the analysis

For the purpose of this analysis, the criteria for patients selection were: (1) age ⩾ 90 years; (2) National Institutes of Health Stroke Scale (NIHSS) score 6 or more; (3) Alberta Stroke Program Early CT score (ASPECTS) 5 or more^17^; (4) LVO sites: distal internal carotid artery, middle cerebral artery segments M1 or M2; (5) initiation of the MT had to be possible within 6h after the stroke onset; (6) pre-event modified Rankin Scale (mRS) score of 0–2. Intravenous thrombolysis (IVT) with intravenous tissue plasminogen activator (tPA) was administered in all patients who presented within 4.5 h of stroke symptom onset and without contraindications according to the guidelines.

### Data collection

All information were collected prospectively, such as medical history, demographic characteristics, history of previous stroke or transient ischemic attack (TIA), baseline NIHSS, admission therapy, site of the occlusion, procedural management and variables, key time points. Level of frailty prior the index event was assessed retrospectively using the Clinical Frailty Scale.^18^ The investigators received training and qualification certificates to record NIHSS and mRS. NIHSS was performed in all patients on admission and 24 h after the MT. The prescription of any antiplatelets and anticoagulant before admission was recorded and included the use of any Direct oral anticoagulant (DOAC) therapy (defined as one of the following drugs and dosages: apixaban 2.5 mg or 5 mg twice daily; dabigatran 110 mg or 150 mg twice daily; edoxaban 30 mg or 60 mg once daily; or rivaroxaban 15 mg or 20 mg once daily); Vitamin K antagonist (VKA) (defined as treatment with acenocoumarol/ warfarin). The choice of treatment was decided by the treating physician as part of routine clinical care pre-admission. The extent of the initial core infarct was determined on pre-therapeutic CT using ASPECTS. In addition, independent raters (consultant neuroradiologists) who did not participate in the endovascular stroke treatment of included patients, evaluated pre-therapeutic CT, and follow-up CT at 24 h. The pre-therapeutic CT was evaluated to assess the collateral score (CS). CS grading was based on the 5-point grading system proposed by Souza et al.^19^ Intracranial CTA maximum intensity projections were used for the grading the CS: 0 = absent collaterals in >50% of an MCA M2 branch (superior or inferior division) territory; 1 = diminished collaterals in >50% of an MCA M2 branch territory; 2 = diminished collaterals in <50% of an MCA M2 branch territory; 3 = collaterals equal to the contralateral hemisphere; and 4 = increased collaterals.

### Study outcomes

The primary outcome was the ordinal distribution of 90-day mRS scores between non-GA and GA. The 90-day mRS was assessed by a stroke neurologist during a clinical follow-up visit or if a clinical visit was not possible in a telephone interview. The secondary clinical study outcomes included 3-month mortality; symptomatic intracranial hemorrhage (sICH) on follow-up imaging, usually obtained 1 day after treatment; symptomatic ICH defined as any ICH on follow-up imaging associated with ⩾4 points worsening in NIHSS occurring within 7 days of acute ischemic stroke^20^; rate of successful recanalization assessed by applying the modified thrombolysis in cerebral infarction (TICI) classification.^21^ Successful recanalization was defined as grade 2b, 2c or 3 of recanalization.

### Statistical analysis

We used inverse probability weighting (IPW) to balance the baseline characteristics of the exposed and unexposed cohorts (GA vs non-GA group) aiming to reduce the confounding factors by indication of anesthesia type on study outcomes. A detailed methodological explanation of the IPW estimation process is available in the Supplemental Methods. In brief, weights were obtained calculating the probability of treatment allocation (i.e. anesthesia type) while controlling for a set of relevant variables that could have influenced the treatment choice. The weights obtained were then used to balance the baseline covariates, therefore creating a pseudo-population independent of the measured confounders (i.e. pseudo-randomization).^22^ A weighted ordinal logistic regression (shift analysis) with a robust estimator explored mRS shift at 90-days in GA versus non-GA treated patients (primary outcome), alongside pre-specified clinical variables of interest (i.e. NIHSS score, use of intravenous thrombolysis and distal occlusion) and relevant residual confounders (i.e. unbalanced variables). Likewise, a weighted logistic regression model was used to explore secondary outcomes. R Studio (ver 2024.4) with the packages *ipw* and *survey* was used for statistical analysis.

## Results

Overall, our cohort 139 patients aged 90 years or older with acute ischemic stroke due to anterior circulation LVO consecutively treated with MT in three thrombectomy capable centers. Sixty-two out of 139 (44.6%) were treated with GA while 77 of 139 (55.4%) without GA. Five patients had a conversion from initial non-GA to GA during the procedure and we assigned those patients to the GA group. Weighted and unweighted results for clinical and neuroradiological baseline characteristics are presented in Tables 1 and 2. Overall, good to acceptable balance was obtained for all major baseline variables of interest except for the ASPECT score on admission. Data regarding the mRS at 90 days after the index event was not available in 11 patients, (2 with non-GA and 9 patients with GA).

**Table 1.** Demographic and clinical characteristics.

	Overall population (⩾90) (*N* = 139)	Non-GA (⩾90) (*N* = 77)	GA (⩾90) (*N* = 62)	SMD unweighted	SMD weighted
Demographics					
Age, years [median (IQR)]	92 (90–93)	92 (90–93)	92 (90–93)	0.01	0.01
Female sex [n, (%)]	45 (32.4)	22 (28.6)	23 (37.1)	0.19	0.03
Hypertension [n, (%)]	91 (65.5)	57 (74.1)	34 (54.8)	0.40	0.09
Diabetes mellitus [n, (%)]	14 (10.1)	7 (9.1)	7 (11.3)	0.08	0.07
Hypercholesterolemia [n, (%)]	54 (38.8)	24 (31.2)	30 (48.4)	0.37	0.02
Atrial fibrillation [n, (%)]	72 (51.8)	47 (61.1)	25 (40.3)	0.40	0.13
Coronary artery disease [n, (%)]	10 (7.2)	4 (5.2)	6 (9.7)	0.18	0.23
Congestive heart failure [n, (%)]	23 (16.5)	10 (12.9)	13 (20.9)	0.22	0.09
Previous TIA/ischemic stroke [n, (%)]	13 (9.4)	9 (11.7)	4 (6.5)	0.18	0.19
Dementia [n, (%)]	2 (1.4)	1 (1.3)	1 (1.6)	0.02	0.06
Malignancy [n, (%)]	24 (17.3)	11 (14.3)	13 (20.1)	0.23	0.19
Admission therapy					
Anticoagulation on admission [n, (%)]	28 (20.1)	20 (26)	8 (12.9)	0.32	0.02
Antiplatelet therapy on admission [n, (%)]	26 (18.7)	19 (24.7)	7 (11.3)	0.34	0.08
NIHSS on admission [median (IQR)]	17 (14–20)	18 (16–21)	16 (13–19)	0.64	0.11
COPD	6 (4.3)	3 (3.8)	3 (4.8)	0.05	0.06
Frailty score (CSF) [mean, sd]	1.90 (0.73)	2.03 (0.74)	1.74 (0.70)	0.37	0.02
Pneumonia	32 (23.0)	16 (20.7)	16 (25.8)	0.12	0.04

NIHSS: national institutes of health stroke scale; SMD: standardized mean difference; TIA: transient ischemic attack; GA: general anesthesia; CSF: clinical frailty scale.

**Table 2.** Neuroradiological and procedural features.

	Overall population (⩾90) (*N* = 139)	Non-GA (⩾90) (*N* = 77)	GA (⩾90) (*N* = 62)	SMD unweighted	SMD weighted
ASPECTS score on admission [median (IQR)]	9 (8–9)	9 (8–10)	8 (8–9)	0.34	0.34
Distal occlusion [n, (%)]	20 (14.4)	9 (11.7)	11 (17.8)	0.18	0.03
Collateral score status				0.01	0.05
0 [n,(%)]	38 (27.3)	17 (22.1)	21 (32.3)		
1 [n,(%)]	51 (36.7)	33 (42.9)	18 (29)		
2 [n,(%)]	43 (31)	23 (30)	20 (32.3)		
3 [n,(%)]	5 (3.6)	3 (3.9)	2 (3.2)		
4 [n,(%)]	2 (1.4)	-	2 (3.2)		
IVT [n, (%)]	102 (73.4)	47 (61)	55 (88.7)	0.72	0.8
Symptom onset to groin puncture time (min), [median (IQR)]	243 (195–303.3)	245 (206.5–307.3)	231 (182–298)	0.31	0.23
Door to groin puncture time (min), [median (IQR)]	111 (59–140)	111.5 (68.8–138.5)	107 (50–144)	0.11	0.06

ASPECTS: the Alberta stroke program early CT score; Distal occlusion: M2: middle cerebral artery segments M2; GA: general anesthesia; IVT: intravenous thrombolysis.

### Primary outcomes

**Figure 1.**
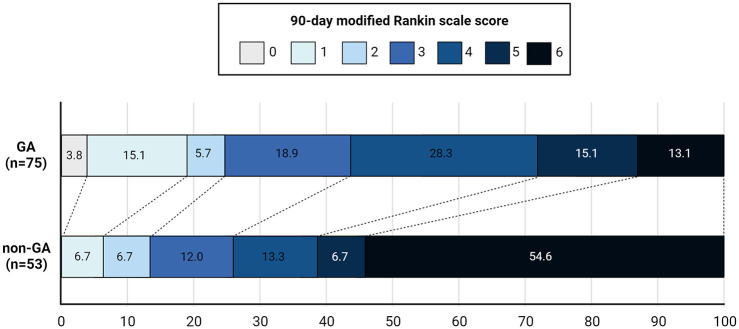
Distribution of modified Rankin Scale (mRS) scores at 90 days in nonagenarian patients treated with general anesthesia (GA) and with non-GA techniques.

The mRS shift analysis showed a significant difference in the 90-day ordinal distribution of mRS scores, with higher mRS in non-GA treated patients (cOR 3.67, CI 95% 1.73–7.80; *p* = 0.001) (Table 3 and Figure 1). When considering the covariates of interest and the unbalanced variables in the model (i.e. ASPECT), non-GA was confirmed as an effect modifier for worse outcome (acOR 2.74, CI 95% 1.11–6.75; *p* = 0.029) alongside NIHSS at baseline (OR 1.28, CI95% 1.12–1.46; *p* = 0.001) (Table 3). In Supplemental Table 1 we reported the 90-day mRS shift analysis reporting non-GA techniques, with higher mRS in patients treated with conscious sedation rather than local anesthesia (cOR 3.76, CI95% 1.69–8.38; *p* = 0.001). When considering in the model the covariates of interest and the unbalanced variables, conscious sedation was confirmed as an effect modifier for worse outcome (acOR 2.94, CI95% 1.25–6.90; *p* = 0.014).

**Table 3.** Weighted shift analysis for mRS at 90 days.*

Predictors	mRS shift (univariate)		
	Common odds ratio	CI	*p*
Non-GA	3.67	1.73–7.80	0.001
	mRS shift (multivariate)		
Predictors	Adjusted common odds ratio	CI	*p*
Non-GA	2.74	1.11–6.75	0.029
IVT	1.24	0.48–3.21	0.661
Distal occlusion	2.16	0.48–9.70	0.313
NIHSS (per unitary increase)	1.28	1.12–1.46	0.001
ASPECT score (>7)	1.22	0.42–3.54	0.713
Sex (female)	0.89	0.33–2.41	0.811
COPD	0.55	0.06–4.89	0.589
Pneumonia	0.82	0.29–2.26	0.693
Frailty score (CSF)	1.70	0.82–3.50	0.151
Atrial fibrillation	0.85	0.31–2.36	0.751
Heart failure	2.82	0.62–12.77	0.176
Coronary artery disease	2.30	0.56–9.49	0.246

* mRS not available in 11 patients (two with non-GA and nine patients with GA).

### Secondary outcomes

The multivariable weighted logistic regression model showed that non-GA was associated with increased likelihood of death at 90 days in patients aged 90 years or older treated with MT for anterior circulation LVO (OR 10.41, 95% CI 2.91–37.22; *p* < 0.001). Contrariwise, non-GA was not an independent predictor of sICH or successful recanalization post MT, respectively (OR 1.90, 95% CI 0.25–14.51; *p* = 0.532) and (OR 1.54, 95% CI 0.51–4.63; *p* = 0.442) ( Supplemental Table 2).

## Discussion

Our multicentre cohort study based on an IPW analysis showed that GA was associated with some clinical advantages over non-GA in patients aged 90 years and older who were treated with MT for anterior circulation LVO. We found a more favorable distribution of mRS scores at 90 days after the index stroke among patients treated with GA versus non-GA. Moreover, our analysis showed that non-GA was an independent predictor of 90-day mortality in nonagenarians treated with MT.

Our study findings are consistent with the recently published literature that suggested that GA is associated with improved functional recovery at 90 days compared to non-GA techniques in patients with ischemic stroke treated with MT.^13^ Indeed, in this subgroup of patients, in line with previous evidence,^23^ our study showed that patients treated with conscious sedation rather than general anesthesia had worse outcome at 90-days as measured on the mRS. Although conscious sedation is associated with potential benefits,^24^ including reduced manpower and time requirements, it has drawn some criticism because of longer procedural time due to the movement of the patients, more exposure to radiation, and a lack of airway control.^25^ Comparable thrombectomy data in the nonagenarian stroke population are sparse in literature. Although this population accounts for a substantial proportion of all stroke patients, elderly patients are often excluded from, or under-represented in, many of stroke clinical trials. Majidi et al. showed the procedural aspects and clinical outcome of MT among 113 octogenarian and nonagenarian acute ischemic stroke patients with a median age of 85 years old.^26^ In their study, only 20% of the patients underwent MT with GA. The authors showed that although the median groin puncture to recanalization time was significantly higher among patients with GA, this was not translated to any significant difference in clinical outcome. However, the authors did not provide data specifically related to the subgroup of patients aged 90 years and older. Meyer et al.^12^ investigated outcome and safety of MT in in patients aged 90 years or older treated with MT for anterior circulation LVO enrolled in the GRS-ET registry (German Stroke Registry-Endovascular Treatment). The authors did not find a significant difference between patients with good versus poor functional outcome at 90 days when compared according to the types of anesthesia. In our current study, 44.6% of our nonagenarian patients undergoing MT were treated with GA while 55.4% with non-GA techniques. Given the observational nature of our study, the type of anesthesia chosen for each procedure, was left to the discretion and experience of the operator and were not dictated by a specific protocol. In this regard, it is noteworthy to mention that we used an IPW analysis to balance the baseline characteristics of the two groups (GA vs non-GA group) aiming to reduce the confounding effect of a potential bias by indication given the retrospective and non-randomized nature of the study.

Another important finding of our study is that non-GA was an independent predictor of 90-day mortality in nonagenarians treated with MT despite taking comorbidities and frailty status into consideration in our analysis. It is important to highlight that our cohort of nonagenarians had a good baseline functional status (mRS 0–2) prior to undergoing MT. Consequently, the overall burden of comorbidities and level of frailty within this group was lower than average. This characteristic allowed for a clearer assessment of the impact of anesthesia type on clinical outcomes, minimizing the confounding effects of severe pre-existing health conditions. The main advantage of non-GA techniques is that they allow for potentially shorter door to puncture times, and the ability to assess patients’ neurologic status immediately after MT.^27^ However, not all patients undergoing MT are suitable for non-GA owing to factors such as patient movement, agitation, or coma. Moreover, past studies in RCTs with highly selected populations have shown that between 2.9% and 20% of patients have been converted from conscious sedation to GA.^28–33^ Conversion to GA is an unscheduled and emergency procedure associated with more prolonged procedural times and poor outcomes, and is likely to be more frequent in unselected, real-world situations.^34^ Planning GA from the start removes this potential risk. The major potential hazards of GA are inadvertent hypotension caused by the induction and maintenance of GA and potential procedural delay.^35,36^ Nevertheless, the advantages of GA include control of respiration and patient movement, therefore, facilitate navigating the catheters through the vascular anatomy and reaching the occlusion site. Therefore, use of GA might play an integral role in improving outcome in this patient population where vessel tortuosity and vascular atherosclerotic disease are common and might pose significant technical challenges to the interventionalists.^7,9^

Our analysis has the following strengths: (1) large cohort of nonagenarian patients; (2) multicentre study; (3) data ascertainment undertaken systematically; (4) the use of an IPW analysis to adjust for the indication bias. Nevertheless, our study also has several limitations. First, the retrospective observational non-randomized design of the study that might have introduced bias, even though we accounted for this using an IPW approach. Secondly, we included patients who had the initiation of the procedure within 6 h after the stroke onset. Data regarding the mRS at 90 days after the index event was not available in 11 patients. Our additional analysis showed how the effect of non-GA on worse outcome is mainly driven by the conscious sedation subgroup, although the limited number of cases in the local anesthesia group (*n* = 5) limits these further interpretations. Finally, our study lacks specific assessment of cognitive function at the 90-day follow-up. While functional outcomes, such as the mRS and mortality rates, were thoroughly evaluated, cognitive status was not directly measured. Cognitive decline can significantly impact quality of life and functional independence, particularly in older populations.^37^ Future studies should consider including comprehensive cognitive assessments to better understand the broader effects of the intervention on patients’ overall neurological health.

## Conclusions

The study indicated that nonagenarian patients with acute ischemic stroke treated with MT without GA have a worse prognosis than their counterparts undergoing GA. Findings and implication of the present study should be interpreted with caution due to the observational design and relatively limited sample. Nevertheless, the topic deserves further investigation to reveal if anesthesia practices can influence prognosis in patients aged 90 and above who are treated with MT for acute ischemic stroke.

## Supplemental Material

sj-docx-1-eso-10.1177_23969873241293009 – Supplemental material for Outcomes of different anesthesia techniques in nonagenarians treated with mechanical thrombectomy for anterior circulation large vessel occlusion: An inverse probability weighting analysis Supplemental material, sj-docx-1-eso-10.1177_23969873241293009 for Outcomes of different anesthesia techniques in nonagenarians treated with mechanical thrombectomy for anterior circulation large vessel occlusion: An inverse probability weighting analysis by Viva Levee, Mariarosaria Valente, Francesco Bax, Liqun Zhang, Simona Sacco, Matteo Foschi, Raffaele Ornello, Katherine Chulack, Emma Marchong, Fahad Sheikh, Feras Fayez, Caterina Del Regno, Mohammed Aggour, Massimo Sponza, Francesco Toraldo, Razan Algazlan, Kyriakos Lobotesis, Daniele Bagatto, Nina Mansoor, Dheeraj Kalladka, Vladimir Gavrilovic, Cristian Deana, Flavio Bassi, Berry Stewart, Gian Luigi Gigli, Soma Banerjee, Giovanni Merlino and Lucio D’Anna in European Stroke Journal

sj-docx-2-eso-10.1177_23969873241293009 – Supplemental material for Outcomes of different anesthesia techniques in nonagenarians treated with mechanical thrombectomy for anterior circulation large vessel occlusion: An inverse probability weighting analysis Supplemental material, sj-docx-2-eso-10.1177_23969873241293009 for Outcomes of different anesthesia techniques in nonagenarians treated with mechanical thrombectomy for anterior circulation large vessel occlusion: An inverse probability weighting analysis by Viva Levee, Mariarosaria Valente, Francesco Bax, Liqun Zhang, Simona Sacco, Matteo Foschi, Raffaele Ornello, Katherine Chulack, Emma Marchong, Fahad Sheikh, Feras Fayez, Caterina Del Regno, Mohammed Aggour, Massimo Sponza, Francesco Toraldo, Razan Algazlan, Kyriakos Lobotesis, Daniele Bagatto, Nina Mansoor, Dheeraj Kalladka, Vladimir Gavrilovic, Cristian Deana, Flavio Bassi, Berry Stewart, Gian Luigi Gigli, Soma Banerjee, Giovanni Merlino and Lucio D’Anna in European Stroke Journal
