# Supplementary material for: Outcomes of different anesthesia techniques in nonagenarians treated with mechanical thrombectomy for anterior circulation large vessel occlusion: An inverse probability weighting analysis
Source: Eur Stroke J. 2024 Oct 30;10(2):379–86. doi: 10.1177/23969873241293009 (PMC11556564; doi:10.1177/23969873241293009)
Supplement: sj-docx-1-eso-10.1177_23969873241293009 – Supplemental material for Outcomes of different anesthesia techniques in nonagenarians treated with mechanical thrombectomy for anterior circulation large vessel occlusion: An inverse probability weighting analysis [file sj-docx-1-eso-10.1177_23969873241293009.docx]

**SUPPLEMENTARY METHODS**

**Inverse probability weighting calculation**

Firstly, we calculated the probability of assignment to non-GA adjusting for a set of pre-defined covariates (i.e. propensity score), specifically: presence of hypertension, atrial fibrillation, heart failure, hypercholesterolemia, chronic obstructive pulmonary disease, distal vessel occlusion, use of intravenous thrombolysis, antiplatelet agents, frailty score, pre-event mRS, and baseline NIHSS. Subsequently, stabilized weights were obtained dividing the crude probability of the observed exposure by the propensity scores, and weight balance was assessed with standardized mean differences (SMD), by considering a value <0.2 an acceptable difference and <0.1 a negligible difference.
