# Supplementary material for: Outcomes of different anesthesia techniques in nonagenarians treated with mechanical thrombectomy for anterior circulation large vessel occlusion: An inverse probability weighting analysis
Source: Eur Stroke J. 2024 Oct 30;10(2):379–86. doi: 10.1177/23969873241293009 (PMC11556564; doi:10.1177/23969873241293009)
Supplement: sj-docx-2-eso-10.1177_23969873241293009 – Supplemental material for Outcomes of different anesthesia techniques in nonagenarians treated with mechanical thrombectomy for anterior circulation large vessel occlusion: An inverse probability weighting analysis [file sj-docx-2-eso-10.1177_23969873241293009.docx]

**Supplemental Table 1. Shift analysis for mRS≥3 at 90 days with reporting of non-GA subgroups (i.e., conscious sedation and local anaesthesia)**

|  | **mRS shift (univariate)** | | |
| --- | --- | --- | --- |
| *Predictors* | *Common Odds Ratio* | *CI* | *p* |
| Non-GA (vs. GA)  Conscious sedation  Local anesthesia | 3.76  0.55 | 1.69 – 8.38  0.17-1.80 | **0.001**  0.317 |
|  | **mRS shift (multivariate)** | | |
| *Predictors* | *Adjusted Common Odds Ratio* | *CI* | *p* |
| Non-GA (vs. GA)  Conscious sedation  Local anesthesia | 2.94  0.49 | 1.25 – 6.90  0.08-3.01 | **0.014**  0.438 |
| i.v. thrombolysis | 1.05 | 0.42 – 2.65 | 0.916 |
| Distal occlusion | 2.06 | 0.57 – 7.41 | 0.264 |
| NIHSS  (per unitary increase) | 1.26 | 1.09 – 1.45 | **0.002** |
| ASPECT score (>7) | 1.46 | 0.47 – 4.49 | 0.508 |
| Sex  (female) | 0.99 | 0.36 – 2.74 | 0.987 |
| COPD | 0.36 | 0.04 – 3.36 | 0.367 |
| Pneumonia | 0.72 | 0.25 – 2.08 | 0.540 |
| Frailty score (CSF) | 1.56 | 0.73 – 3.35 | 0.253 |
| Atrial fibrillation | 1.01 | 0.38 – 2.73 | 0.978 |
| Heart failure | 2.81 | 0.52 – 15.19 | 0.228 |
| Coronary artery disease | 1.13 | 0.27 – 4.72 | 0.864 |

**Supplemental Table 2. Weighted logistic regression analysis for mRS≥3 at 90 days, mortality at 90 days and sICH at 24 hours.**

|  | **mRS ≥3 at 90 days** | | | **Mortality at 90 days** | | | **sICH at 24 hours** | | | **TICI**  **post-intervention** | | |
| --- | --- | --- | --- | --- | --- | --- | --- | --- | --- | --- | --- | --- |
| *Predictors* | *Odds Ratios* | *CI* | *p* | *Odds Ratios* | *CI* | *p* | *Odds Ratios* | *CI* | *p* | *Odds Ratios* | *CI* | *p* |
| **Non-GA** | 1.57 | 0.43 – 5.67 | 0.492 | 10.41 | 2.91 – 37.22 | **<0.001** | 1.90 | 0.25 – 14.51 | 0.532 | 1.54 | 0.51 – 4.63 | 0.442 |
| **IVT** | 0.45 | 0.07 – 2.88 | 0.394 | 1.93 | 0.52 – 7.14 | 0.323 | 0.92 | 0.10 – 8.13 | 0.941 | 0.59 | 0.15 – 2.29 | 0.440 |
| **Distal LVO occlusion** | 0.61 | 0.12 – 2.97 | 0.532 | 8.75 | 1.42 – 53.81 | **0.020** | - | - | - | 0.83 | 0.18 – 3.79 | 0.813 |
| **NIHSS on admission**  **(per unitary increase)** | 1.25 | 1.06 – 1.48 | **0.010** | 1.29 | 1.07 – 1.54 | **0.007** | 0.92 | 0.77 – 1.09 | 0.333 | 0.98 | 0.88 – 1.09 | 0.728 |
| **ASPECT score on admission** | 0.35 | 0.12 – 1.06 | 0.064 | 1.12 | 0.64 – 1.94 | 0.692 | 1.02 | 0.50 – 2.07 | 0.962 | 1.14 | 0.74 – 1.76 | 0.562 |
| **Sex (female)** | 0.49 | 0.12 – 1.98 | 0.310 | 0.32 | 0.10 – 1.02 | 0.055 | 4.67 | 0.84 – 25.90 | 0.077 | 0.56 | 0.12 – 2.54 | 0.445 |
| **COPD** | 0.33 | 0.02 – 6.77 | 0.466 | 0.47 | 0.07 – 3.26 | 0.439 | - | - | - | 0.35 | 0.05 – 2.45 | 0.284 |
| **Pneumonia** | 0.86 | 0.13 – 5.64 | 0.870 | 0.41 | 0.13 – 1.25 | 0.114 | 0.42 | 0.06 – 2.88 | 0.373 | 1.31 | 0.28 – 6.13 | 0.729 |
| **Frailty score (CSF)** | 2.91 | 1.06 – 8.00 | **0.038** | 2.40 | 1.07 – 5.38 | **0.034** | 0.88 | 0.30 – 2.61 | 0.813 | 0.65 | 0.31 – 1.37 | 0.255 |
| **Atrial fibrillation** | 1.87 | 0.45 – 7.80 | 0.388 | 0.44 | 0.14 – 1.36 | 0.151 | 0.21 | 0.04 – 1.15 | 0.072 | 1.55 | 0.50 – 4.77 | 0.445 |
| **Heart failure** | 6.30 | 0.55 – 71.92 | 0.137 | 5.70 | 0.92 – 35.39 | 0.062 | - | - | - | 1.24 | 0.20 – 7.69 | 0.815 |
| **Coronary artery disease** | 0.23 | 0.02 – 2.31 | 0.207 | 5.78 | 0.50 – 67.54 | 0.160 | - | - | - | - | - | - |
| R^2^ / R^2^ adjusted | 0.333 / 0.254 | | | 0.368 / 0.290 | | | 0.119 / 0.050 | | | 0.062 / -0.039 | | |

**Legend:** ASPECTS = The Alberta Stroke Program Early CT Score; CSF= Clinical Frailty Scale.; Distal occlusion: M2 = middle cerebral artery segments M2; GA= general anaesthesia; IVT= intravenous thrombolysis; NIHSS = National Institutes of Health Stroke Scale.

**Supplemental Table 3. Outcome reporting for mRS≥3 at 90 days, mortality at 90 days and sICH at 24 hours according to anaesthesia type: non GA (i.e., conscious sedation and local anaesthesia) and GA**

| Anesthesia type | | **mRS ≥3 at 90 days** | **Mortality at 90 days** | **sICH at 24 hours** | **TICI**  **post-intervention** |
| --- | --- | --- | --- | --- | --- |
| Non-GA | Conscious sedation | 78 (74%) | 45 (94%) | 2 (22%) | 26 (30%) |
|  | Local anesthesia | 5 (5%) | 0 (0%) | 0 (0%) | 79 (68%) |
| GA | General anesthesia | 23 (22%) | 3 (6%) | 7 (78%) | 8 (7%) |
